# Experimental Demonstration of Light Focusing Enabled by Monolithic High-Contrast Grating Mirrors

**DOI:** 10.1021/acsami.1c04871

**Published:** 2021-05-19

**Authors:** Paulina Komar, Marcin Gȩbski, James A. Lott, Tomasz Czyszanowski, Michał Wasiak

**Affiliations:** † Institute of Physics, Lodz University of Technology, Wólczańska 219, 90-924 Łódź, Poland; ‡ Institute of Solid State Physics and Center of Nanophotonics, Technical University Berlin, Hardenbergstraße 36, 10623 Berlin, Germany

**Keywords:** MHCG, high-contrast gratings, focusing mirrors, metasurfaces, GaAs

## Abstract

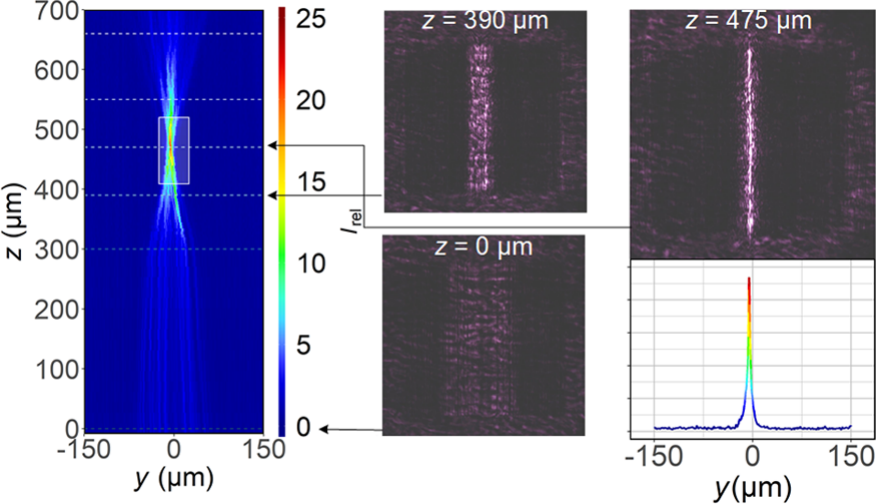

We present the first experimental demonstration of a planar focusing monolithic subwavelength grating mirror. The grating is formed on the surface of GaAs and focuses 980 nm light in one dimension on the high-refractive-index side of the mirror. According to our measurements, the focal length is 475 μm (300 μm of which is GaAs) and the numerical aperture is 0.52. The intensity of the light at the focal point is 23 times larger than that of the incident light. To the best of our knowledge, this is the highest value reported for a grating mirror. Moreover, the full width at half-maximum (FWHM) at the focal point is only 3.9 μm, which is the smallest reported value for a grating mirror. All of the measured parameters are close to or very close to the theoretically predicted values. Our realization of a sophisticated design of a focusing monolithic subwavelength grating opens a new avenue to technologically simple fabrication of the gratings for use in diverse optoelectronic materials and applications.

## 1 Introduction

The progressive miniaturization of optoelectronics and integrated optics is spurring the search for low-dimensional alternatives to conventional focusing lenses and mirrors.^1,2^ The properties of conventional focusing elements are based on their precisely shaped surfaces. When such elements are very large or very small, their fabrication becomes challenging and costly, especially if the focal length is short relative to the diameter. Obtaining a properly shaped mirror or lens of the scale and dimensions normally required for optolectronics and integrated optics is extremely difficult and requires very sophisticated technology.

As an alternative, flat structures with similar properties can be used: diffractive elements^3^ and metasurfaces in the form of subwavelength grating (SG) structures.^4^ In both cases, the focusing mechanism involves locally induced phase changes of the transmitted light, which reproduce the phase profile and enable constructive interference at the focal point. However, the mechanism of phase induction is different. In the case of diffractive lenses (also called Fresnel lenses), the spatial phase delay profile is produced by coaxial radial zones, each of which reproduces part of the surface of a conventional lens. These zones modify the phase of the transmitted light within the range of 2π, by inducing different optical paths.^5^ This imposes wavelength-comparable heights on the elements. As a result, Fresnel lenses may be very thin but are still based on precisely formed curvatures. In the case of an SG, the maximum height of the element for the same 2π range of phase change can be as little as a quarter-wavelength. Such lenses are composed of subwavelength elements, usually rectangular in cross section, arranged in one- or two-dimensional gratings. The interaction of the elements with the incident light modifies the phase of the light, by coupling the nonzero diffraction orders to the modes propagating in the lateral direction. Those modes can be scattered by the subwavelength elements out of the grating into the zeroth diffraction order with a specific phase.^6^ There is still some debate over which structure is superior,^7−9^ as their properties are comparable. Nonetheless, SGs can be fabricated in a relatively simple process, requiring single-step lithography,^8^ whereas the fabrication of diffraction lenses requires multiple steps.^10^

The challenges posed by the fabrication of submillimeter focusing mirrors are similar to those related to the manufacture of lenses, but in addition, high reflectivity must be ensured. Traditionally, this is achieved by covering the curved surface with a metal or multilayer periodic structure. Subwavelength gratings allow for very high power reflectance and simultaneously enable a wide range of variation in the phase of the reflected light.^1,11,12^ These two advantages make it possible to create highly reflective focusing (or otherwise directing) flat SG mirrors.

There are few reports in the literature on the fabrication of SG focusing mirrors. The first, composed of a silicon grating deposited on a quartz substrate, was demonstrated by Fattal et al.^13^ This mirror had a focal length of almost 20 mm and a 1/*e*^2^ spot diameter of ∼300 μm. No information was given on the intensity of light at the focal point. Later on, a grating from amorphous silicon on a glass substrate designed for 980 nm was investigated by Klemm et al.^14^ The focal lengths of the investigated mirrors were between 60 and 140 μm, and the spot diameter was 5 μm. The light intensity at the focal point in a reflection configuration was 3–4 times larger than in the back focal plane (transmitted light); however, these numbers were not referenced to the incident light intensity. In 2016 and 2017, Fang et al.^15,16^ demonstrated mirrors in the form of a Si grating on SiO_2_ cladding, with a Si substrate. The focal lengths of the mirrors were around 11 mm, and the intensity at the focal point was approximately 1.2 of the intensity of the incident light. In all four of these constructions, the incident and reflected light propagated in air. In another mirror, reported by Chen et al.,^17^ the incident and reflected light propagated in InP and the grating was made of Si. However, as in the previous cases, InP has a lower refractive index than the grating. In ref (17), the focal length was around 400 μm. No information was given on the light intensity at the focal point relative to the incident light intensity. To the best of our knowledge, these five constructions are the only focusing mirrors that have been reported so far. A different type of reflecting, although not focusing, metasurface was presented in ref (18). Essentially, it is a transmissive metasurface placed on a flat gold mirror. This combination acts as a mirror that modifies the phase of the reflected wave in a specific manner. In total, four different materials are used to form this mirror, so it is not a monolithic structure.

Our construction differs from the focusing mirrors that have been reported previously in three crucial respects. First, the mirror is composed of a monolithic high-contrast grating (MHCG) formed on the surface of a GaAs wafer. The MHCG does not require a low-refractive-index region beneath the ridges, so it is much more versatile than more conventional mirrors. Second, the light is reflected back into the highest-refractive-index medium (GaAs). This is the most preferable configuration if the mirror is to be used to focus light within an optoelectronic device. However, this configuration requires cancellation of higher diffraction orders that normally are not suppressed in the high-refractive-index region and cause reduction of focusing ability. Third, our mirror focuses light in one direction rather than two directions (i.e., the focus is in the form of a line, not of a disk). Nonetheless, the light intensity at the focal point shown in this article is over an order of magnitude higher than any reported for a focusing mirror previously in the literature.^15,16^ Out of five reports on focusing grating mirrors,^13−17^ only two^15,16^ provide information on the light intensity relative to the incident light intensity at the focal point, which is 1.2 at best.

Conventional SGs are high-refractive-index gratings. They may be suspended in air^19^ or implemented on a low-refractive-index layer.^20^ This is typically a dielectric layer, which makes epitaxial growth fairly complex and also limits the application of SGs in electrical devices. An MHCG is a simple version of an SG. It takes the form of a surface relief on a transparent substance with a sufficiently high refractive index (≳1.75). An MHCG can provide 100% power reflectance with a relatively large high-reflection stopband.^12,21^ Monolithic high-contrast grating mirrors are inherently robust and resistant to mechanical damage. Their parameters can be precisely controlled by standard electron beam lithography (EBL) or can be mass produced by nanoimprint lithography (NiL).^22^ A given MHCG may also cover an arbitrarily large surface area—in contrast to large SGs suspended in air, which are fragile and highly susceptible to collapse.^23^ MHCGs enable manipulation of the reflected wave phase in a similar way to conventional SGs. The same rules therefore apply to the construction of an MHCG focusing mirror as have been described in ref (11).

## 2 Results and Discussion

**Figure 1:**
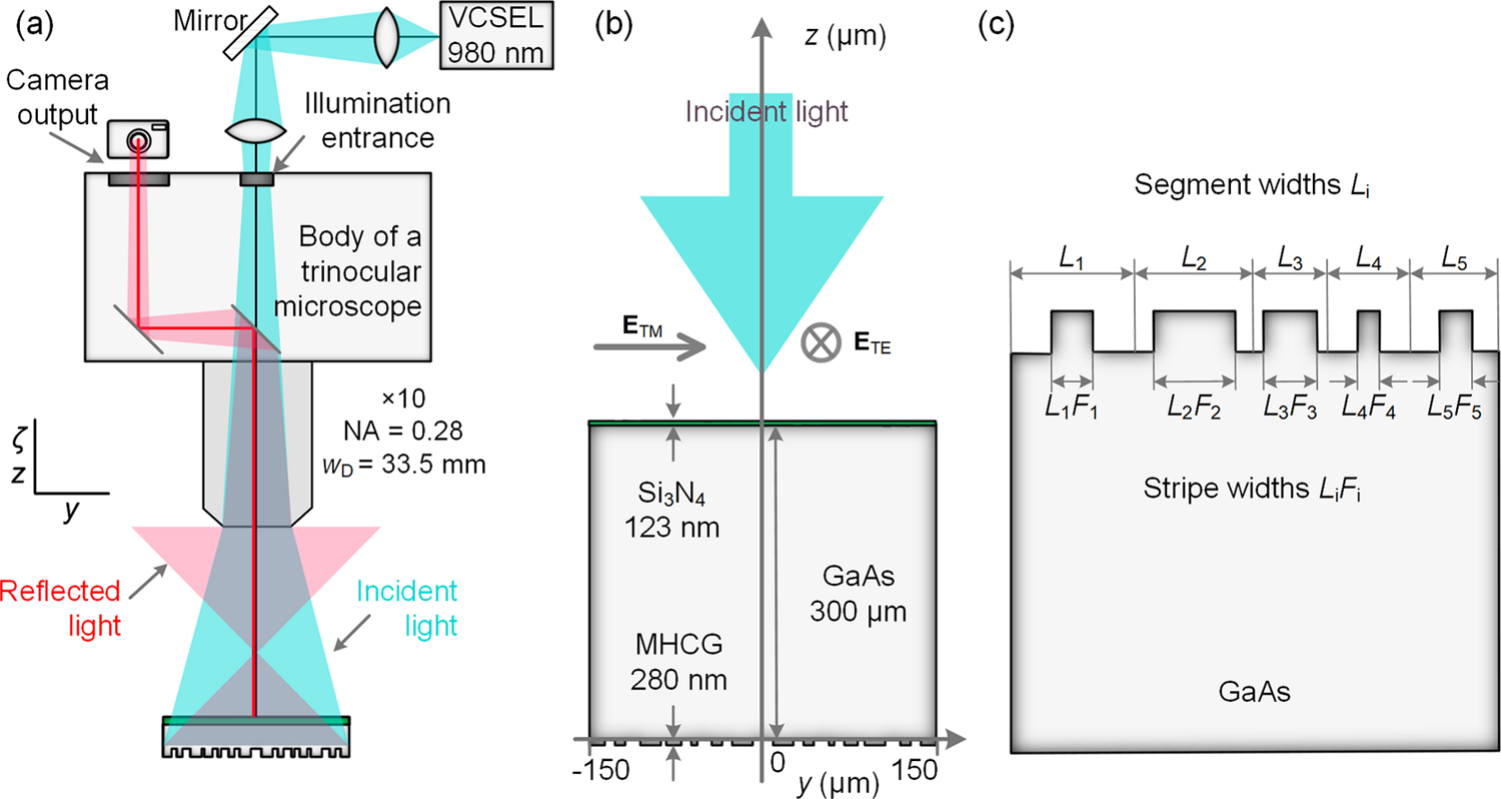
(a) Scheme of the measurement setup and (b) zoomed image showing the configuration of the focusing grating mirror under the microscope lens. NA is the numerical aperture and *w*_D_ is the working distance of the objective. (c) Scheme of a piece of a nonperiodic MHCG.

Here, we investigate an MHCG that acts as a planar reflector, focusing light in one direction. The focusing mirror was designed according to a procedure described in detail elsewhere.^11^ The GaAs (single layer) grating is 280 nm thick, and its lateral dimensions are 300 μm × 300 μm. It was manufactured on the surface of a 300 μm thick GaAs substrate (for details on the fabrication procedure, see Section 4.1). As schematically shown in Figure 1c, the focusing grating mirror is a nonperiodic structure in which segment widths *L*, stripe widths *LF*, and fill factors *F* (a ratio between a stripe width and a segment width) vary. In the investigated mirror, the grating segment widths range between 0.385 and 0.98 μm, the fill factors range between 0.28 and 0.635, and the stripe widths range between 0.175 and 0.415 μm.

**Figure 2:**
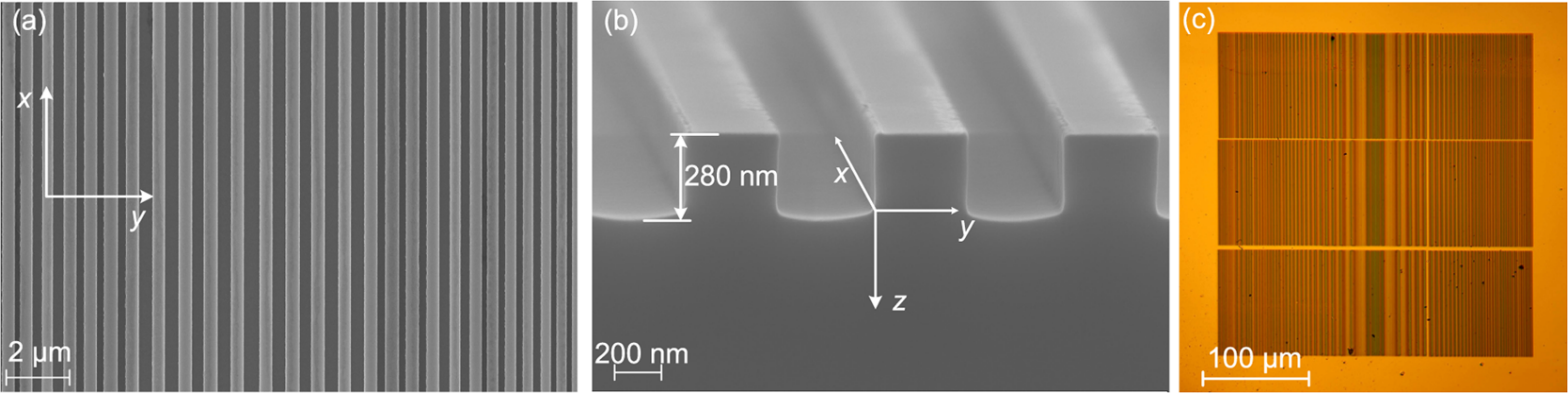
Scanning electron microscope images: (a) top view of the focusing grating mirror and (b) perspective view on the cross section of the test grating fabricated under conditions identical to the focusing grating mirror studied here. (c) Optical microscope photograph of the fabricated focusing grating.

As can be seen in the scanning electron microscope images shown in Figure 2a,b, we were able to fabricate grating stripes that exhibit a very high degree of uniformity along the *x* axis, with very smooth edges and steep sidewalls. While the top-view image shown in Figure 2a was taken for the focusing grating mirror investigated here, the perspective-view image visible in Figure 2b was acquired for a test sample fabricated under identical conditions. Nonetheless, as our fabrication conditions were very stable and the process was highly reproducible, we expect that the cross section of the actually studied focusing grating mirror has comparable quality to the test sample. The small gaps visible in Figure 2c emerged due to an inaccuracy of up to 5 μm in the positioning of a motorized translation stage between the write-fields during lithography. We will show that despite this slight defect, the fabricated planar mirror reflects and focuses light almost in accordance with the simulations.

**Figure 3:**
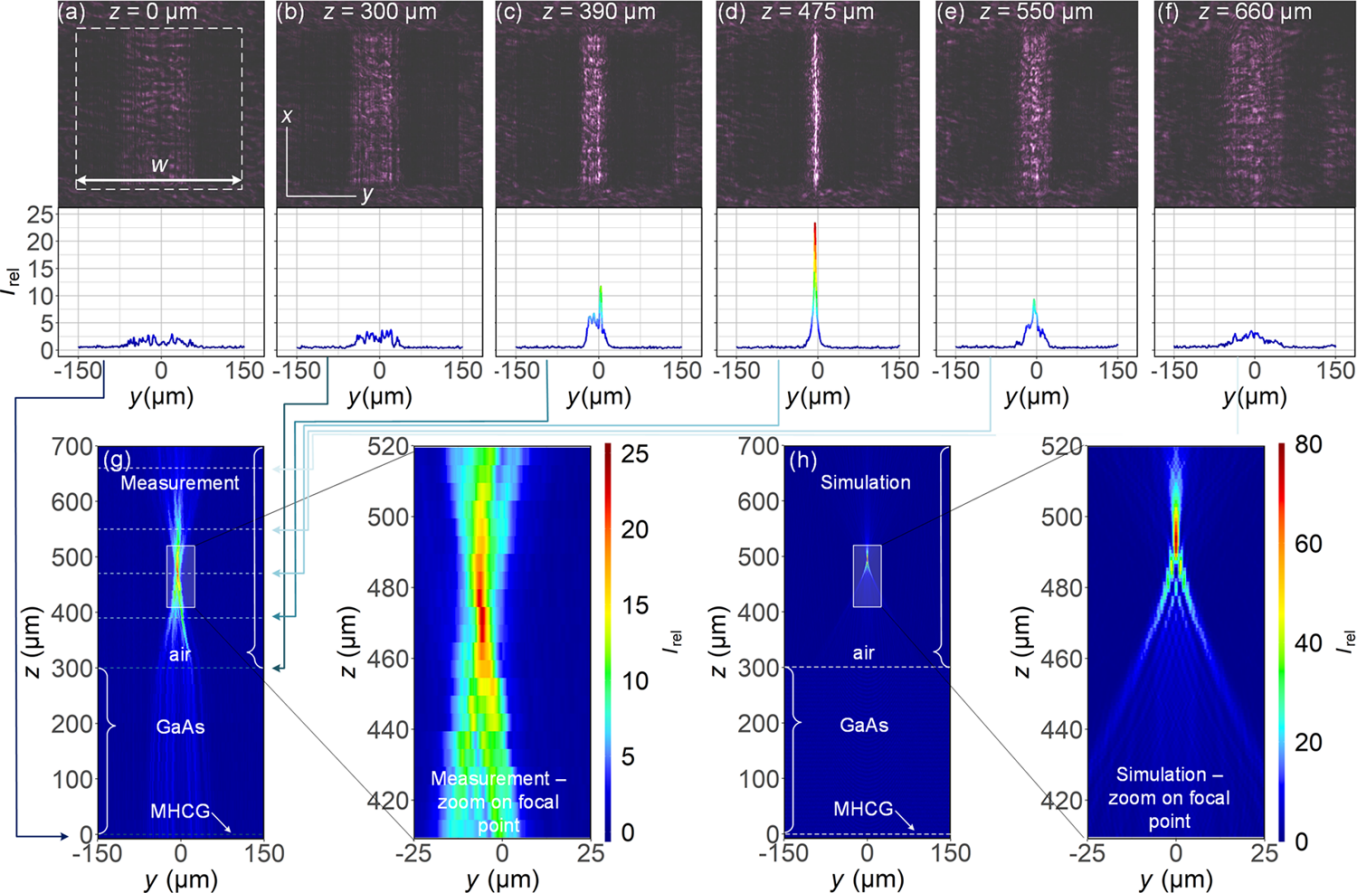
(a–f) Photos taken by a camera at various heights above the grating and the corresponding intensity profiles (color scale is identical to the scale in (g); for details on the reconstruction procedure, see Section 4.2). Maps of the light intensity (g) reconstructed from the photographs and (h) from simulations.

We investigated the mirror using the setup shown schematically in Figure 1a and discussed in Section 4.2. As presented in Figure 1b, the mirror was placed on a microscope stage with the grating on the underside. The incident light was linearly polarized with the nonzero component of the electric field perpendicular to the stripes of the grating (TM polarization), as indicated in Figure 1b. The incident light is not strictly perpendicular to the grating surface (as was assumed in our simulations), because it passes through the microscope lens. However, since the numerical aperture of the mirror is much higher than that of the objective (see Section 4.3 for details), this effect does not have a significant impact on the results. In the following, *z* denotes vertical positions above the grating, while ζ is used to indicate the vertical positions of the microscope head. The focusing of the reflected light was studied by moving the microscope head, with a digital camera attached, along the ζ axis. At the lowest position of the head (ζ = 0 μm), the image was focused on the surface of the mirror, i.e., at the bottom of the sample. As the microscope head with the camera was moved upward, a narrowing of the reflected light was initially observed, as can be seen in Figure 3a–d. Further increases in ζ led to a gradual widening of the image of the reflected light (see Figure 3e, f). The symbol *I*_rel_ denotes the intensity of light relative to the intensity of the incident light, averaged over *x*, i.e., over the direction in which the grating is nominally homogeneous. Simply by performing such a qualitative analysis, we were able to determine that the focal point of the mirror was at ζ = 260 μm. Because the reflected light travels first through roughly 300 μm of GaAs with a refractive index of 3.5, the microscope image is focused on the surface of the sample at ζ = 85 μm. For a better demonstration of the focusing effect, see the video in the Supporting Information.

The intensity profiles presented in Figure 3a–f were determined using the procedure described in Section 4.3. The photographs used to reconstruct the light intensity were taken with a 5 μm step in the vertical position of the microscope head. In the reconstructed light intensity map presented in Figure 3g, the vertical coordinate (*z*) is determined from ζ by taking into account the refractive index of GaAs. As can be seen in Figure 3, there is very good agreement between the maps from the experiment and those from our simulations. The simulations were performed using the plane-wave reflection transformation method discussed in detail in ref (24). The simulated and measured maps determined for the same grating mirror but perpendicular polarization (transverse electric—in which the electric field is parallel to the stripes of the grating) are shown in Figure S1 in the Supporting Information. According to simulations, no light focusing is expected to occur. Our experiments show that the focusing is not completely absent; however, the focal point is not well defined, but elongated.

The focal length of the grating mirror was determined experimentally as 475 μm, which is very close to the designed value of 493 μm. The slight horizontal shift in the focal point from *y* = 0 μm to about −5 μm was presumably caused by the nonideal joint of the writing fields, as discussed in Section 4.1 and visible in Figure 2c. The light intensity at the focal point is smaller than predicted theoretically. However, it is still 23 times larger than the intensity of the incident light, which to the best of our knowledge gives our design the highest light intensity at the focal point that has been reported so far among subwavelength grating mirrors.^15,16^

**Figure 4:**
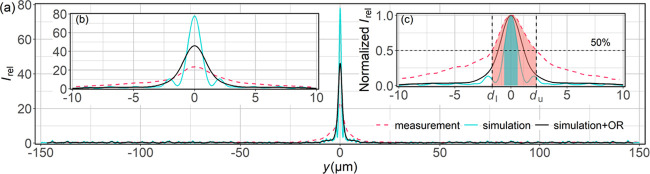
(a) Comparison of the measured and simulated light intensity distributions at the focal point along the entire mirror and (b) close-up of the vicinity of the peak. (c) Normalized light intensity distributions around the peak vicinity. The shaded areas indicate peak widths determined based on FWHM. Red and blue shades correspond to the measurements and simulations. The simulation + OR curve is discussed in the main text.

To perform a deeper analysis of the focusing properties of our mirror, we compared the measured width of the beam at the focal point and the measured focusing efficiency with the simulations. There exist several methods to determine the peak width, and the results strongly depend on the method used.^25^ Here, we compare full width at half-maximum (FWHM) peak widths. Our analysis is based on the intensity profiles along *y* at *z* = 493 μm for the simulated data and at *z* = 475 μm for the measured data, i.e., at the corresponding focal points. The peak position of the measured data was aligned to *y* = 0 μm. Because our optical system has a limited resolution, mainly due to the resolution of the objective (OR), we also calculated a third curve, which is a convolution of the simulated profile with an appropriate Gaussian distribution. This third curve is denoted in Figure 4 as simulation + OR and indicates the measurement limit when using our objective. The limited resolution of the objective leads to a decrease in the light intensity, a widening of the peak, and the disappearance of the oscillations visible in the nondisturbed simulations. A similar, yet even stronger, effect is apparent in the measured curve. As can be seen in Figure 4, both the OR simulated data and the measured data form an outline over the simulated oscillations. However, the experimentally determined light intensity is 50% lower than that obtained when we take into account the OR of the system. We are not sure of the main reason for this discrepancy. It may be due to a combination of overestimating the OR of the system, defects in the fabricated mirror, or the fact that the incident beam was convergent, not parallel.

The magnification of the normalized light intensity in the vicinity of the peak is shown in Figure 4c, where the shaded areas indicate the part of the distribution for which the intensity is larger than 50%. The corresponding FWHM values are 3.9 and 1.3 μm for the measurements and simulations, respectively. Based on the peak widths, we calculated the focusing efficiency η using the following formula
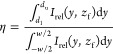
1where *z*_f_ is the vertical coordinate of the position of the focal point, *d*_l_ and *d*_u_ are as defined in Figure 4c, and *w* is the width of the mirror, as shown in Figure 3a. The focusing efficiency values are presented in Table 1. According to the measurements, 23% of the total light intensity is concentrated at the focal point.

**Table 1:** Summary of the Quantities Characterizing the Focusing Properties of the Simulated and Measured Mirrors^a^

	*I*_rel_	*f* (μm)	FWHM (μm)	η (%)	ratio FWHM/λ
simulated	78	493	1.3	34	1.3
simulated + OR	46	493	2.2	35	2.2
measured	23	475	3.9	23	4.0

a *I*_rel_ stands for light intensity relative to the incident light intensity, *f* is the focal length, FWHM is the width of the peak at the focal point, and η is the focusing efficiency.

Finally, we attempted to estimate the reflectance of the mirror, which is a nontrivial problem. Because the numerical aperture of the grating mirror (0.52) is much larger than the numerical aperture of the microscope objective (0.28), our setup is not able to collect all of the light reflected by the grating mirror, as is shown schematically in Figure 1a. To estimate the total amount of reflected light, we used the procedure described in Section 4.3. The approximate measured value for reflectance is *R*_est_ = 0.7, whereas the value according to the simulations is *R*_sim_ = 0.75. Because of the high uncertainty of our estimate, we can only conclude that the reflectance of the investigated mirror is, as expected, very high, and comparable to the simulation.

**Figure 5:**
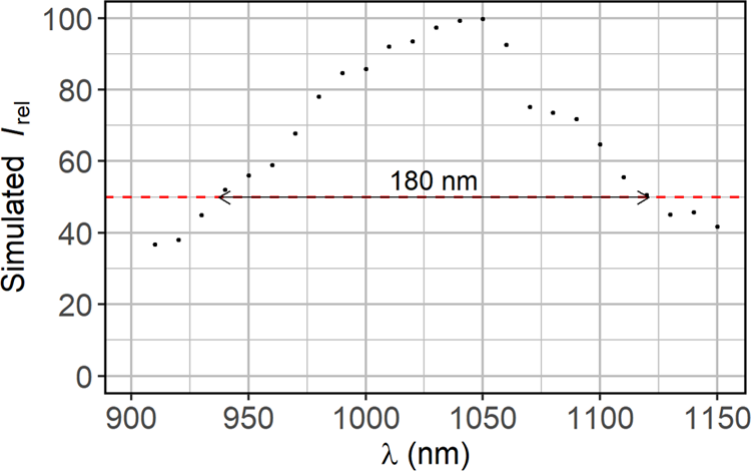
Spectral dependence of the reflected light intensity at the focal point relative to the incident light intensity. Simulations were performed for the focusing grating mirror designed for 980 nm. The red dashed line indicates the level of 50% of maximum *I*_rel_(λ), which was used to determine the bandwidth of the focusing mirror.

To determine the spectral width of the studied focusing grating mirror, we performed numerical simulations in which we analyzed the maximum light intensity at the focal point (*I*_rel_) as a function of the wavelength between 900 and 1150 nm every 10 nm. The light intensity at the focal point relative to the incident light intensity, shown in Figure 5, is not the highest for 980 nm, but for 1050 nm. When we estimate the spectral width as a range of wavelengths for which the light intensity is larger than 50% of the maximum light intensity in the entire studied range of wavelengths (i.e., for λ = 1050 nm), the bandwidth is 180 nm. Thus, it can be concluded that the focusing grating mirror exhibits a broad band rather than wavelength-selective operation.

## 3 Conclusions

In this article, we provide an experimental demonstration of light focusing by a planar focusing mirror based on an MHCG. A 280 nm thick grating mirror (composed solely of GaAs on an underlying GaAs bulk layer) with an area of 300 μm × 300 μm was designed to focus TM-polarized light in a single direction. It was optimized to work for a wavelength of 980 nm under normal incidence and was fabricated using electron beam lithography (EBL) followed by inductively coupled plasma reactive ion etching (ICP-RIE). The measured focal length of the mirror is in very good agreement with the designed value. Moreover, the measured light intensity at the focal point is 23 times higher than the intensity of the incident light. This is about 29% of the simulated value and, to the best of our knowledge, by far the highest value reported so far in the literature for any subwavelength grating-based mirror or lens.^15,16^

As we have described in ref (11), we study planar focusing mirrors that reflect light into the high-refractive-index material, instead of the air. Designing this type of mirror is very challenging, as in principle the reflected light may be not only of the zero order but also of unfavorable higher diffraction orders. To eliminate these higher diffraction orders, the geometrical dimensions of the grating must fulfill particular conditions.

According to our simulations, the grating presented here does not produce noticeable higher diffraction orders. These orders, if present, would not appear in our experiment, because they would be subject to total internal reflection on the GaAs–air interface. Because of the very good agreement between our simulations and the experimental results, we are convinced that the higher orders are not present in the light reflected by the grating mirror. This is an important conclusion, because it means that the proposed grating is also capable of focusing light within GaAs, and therefore within a semiconductor device. Such focusing inside a high-refractive-index material opens the possibility for important new applications, such as in detectors, integrated optics, or high-quality factor cavities with focusing mirrors, as well as perhaps many others that lie beyond the horizon of our imagination.

The very good performance of the presented mirror would not be possible if the phase and intensity of the reflected light were not controlled with a very good accuracy by the monolithic grating. Thus, we suggest that monolithic SGs are not inferior in terms of performance to more conventional SGs with a low-refractive-index cladding. From the technological point of view, as well as from the point of view of applications in electrically driven devices, monolithic structures present many advantages. We believe that monolithic SGs will become the most widely used type of SG in the majority of current and future applications.

## 4 Materials and Methods

### 4.1 Fabrication of Samples

The MHCG focusing reflector was patterned on top of a GaAs substrate using electron beam lithography (EBL). First, the sample was covered with approximately 200 nm thick AR.P6200.09 EBL resist. Next, nine fields 100 μm × 100 μm in size were patterned in a 3 × 3 matrix using a Raith ELPHY Plus EBL tool. The pattern was then etched in Cl_2_ + BCl_3_ + Ar plasma using an inductively coupled plasma reactive ion etching (ICP-RIE) reactor. Finally, the underside of the substrate (the side opposite to the grating) was mechanically polished using a mixture of sapphire powder and distilled water and covered with a 123 nm thick Si_3_N_4_ antireflecting coating by means of plasma-enhanced chemical vapor deposition (PECVD).

### 4.2 Focusing Measurements

For the measurements, the sample was turned upside down so that the grating was on the bottom and the antireflecting coating was on the top. Measurements of the focusing properties were performed by means of a trinocular Motic PSM1000 microscope with an ELWD PLAN APO objective (10× magnification, NA = 0.28, working distance *w*_D_ = 33.5 mm). The microscope has an entrance for an external light source that allows for through-objective illumination of the sample. As the light source, we used a vertical-cavity surface-emitting laser that emits at 980 nm. As shown schematically in Figure 1a, the laser beam was shaped by convex lenses and then directed to the microscope entrance by a mirror.

During the measurements, the internal iris of the microscope was set to its minimum size. In this way, most of the diverging rays of incident light were eliminated and, moreover, only the grating and its nearest surroundings were illuminated. The light reflected by the focusing grating mirror was collected by a digital camera (Sony α6000), the infrared filter of which had been removed from the matrix, attached to the camera output of the microscope (see the scheme in Figure 1a). To determine the focal point of the focusing mirror and the light distribution map, photographs were taken at evenly spaced distances above the grating along the vertical axis. The microscope head with the camera attached was moved upward, while the stage on which the sample was positioned remained stationary. The resolution of the focusing knob, and thus the movement of the microscope in the vertical direction, was 1 μm, and the measurements were taken every 5 μm. All of the photographs were taken with the camera matrix sensitivity set to ISO 100 and with a constant exposure time. They were then saved in ARW format (RAW format for Sony cameras) with a 24 MP resolution. Based on the total size of the mirror, we determined that one pixel on the photograph corresponded to 0.38 μm on the sample, which is in very good agreement with the expected outcome, taking into account the 10-fold magnification of the objective and a pixel size on the matrix of the camera equal to 3.92 μm. The shutter speed was chosen such that all R, G, and B color components were not saturated at the focal point, where the local brightness of the image is highest.

The measurable light intensity profiles were limited by the resolution of our measurement system, i.e., by the resolution of the microscope objective and the camera matrix. To take this effect into account, we convoluted the simulated ideal profiles with a Gaussian distribution. The variance of the distribution is the sum of the variance of the distribution describing the impact of the objective and the variance resulting from the size of the matrix pixel. A detailed discussion on the resolution of the microscope-camera system is given in the Supporting Information. The resulting standard deviation σ ≈ 0.744 μm.

### 4.3 Quantification of Optical Power Reflectance and Light Intensity

The focusing properties were quantified based on the grayscale intensity of the acquired RAW images. The analysis was performed in R language using the *imager* library. To convert the photographs from the RGB format to the grayscale, the function called *grayscale* was used, in which *Luma* was set as the conversion method. In this method, luminance *Y* is linearly approximated by the following formula: *Y* = 0.299·R + 0. 587·G + 0.114·B.

First, as shown schematically by the dashed lines in Figure 3a, the position of the grating was located in a photograph taken at ζ = 0 μm (i.e., when the image was focused on the mirror surface). Then, for each ζ, grayscale intensity profiles *p*_ζ,*x*_(*y*) were extracted of 301 evenly distributed *x* positions, as well as for *y* across the entire mirror width. These were averaged over *x*, obtaining one intensity profile *p̅*_ζ_(*y*) for each image. Finally, to estimate the intensity of the reflected light relative to that of the incident light (*I*_rel_), we measured the intensity of light reflected from the GaAs surface without a grating and used it as a benchmark. We calculated the reflectivity of the GaAs layer covered with the Si_3_N_4_ antireflecting coating and obtained *R*_GaAs+AR_ = 0.336. Additionally, due to the large mismatch between the numerical aperture of the grating mirror under investigation (NA_m_ = 0.52) and the numerical aperture of the microscope objective (NA_o_ = 0.28), a correction factor was introduced. The reason this factor was necessary is shown schematically in Figure 1a, where it can be seen that not all rays diverging from the focus of the mirror were collected by the objective. As the divergence of the beam reflected by the focusing grating is modified only in one dimension, the factor was set to NA_m_/NA_o_ = 1.86 (if the mirror would focus light in two dimensions, the correction factor would be (NA_m_/NA_o_)^2^). In the perpendicular direction, all of the reflected rays will reach the objective (because they come from the objective and reflection angle is equal to the incidence angle), so only one-dimensional (1D) correction is necessary. The angles of incidence of the rays inside the GaAs did not exceed the total reflection limit and the top surface was covered with an antireflecting layer, so we assume that no significant energy was dispersed in the GaAs layer.

## Supporting Information Available

Animation showing how the reflected light is shaped when the microscope head with a digital camera attached to it is moved upward (i.e., further and further away from the grating); It was created based on evenly taken images, and the vertical distance from the grating (*z*) is indicated in the top left corner (MP4)
Simulated and measured maps of the reflected light intensity relative to the incident (TE-polarized) light intensity for the grating designed for TM polarization (Figure S1); photograph of the setup used to determine the range of the linearity of the camera matrix (Figure S2); photograph taken by the camera arranged in the setup shown in Figure S2 indicating the brightest pixel (Figure S3); graph showing the grayscale pixel intensity and all color components (red, green, and blue) intensities of the brightest pixel (Figure S4); magnification of the gray-shaded area from Figure S4 (Figure S5); and red, green, blue, and grayscale pixel intensities of the mean profile extracted from the photograph of the light intensity at the focal point of the investigated focusing grating mirror (Figure S6) (PDF)

## References

[ref1] Chang-Hasnain C. J.; Yang W. High-Contrast Gratings for Integrated Optoelectronics. Adv. Opt. Photonics 2012, 4, 379–440. 10.1364/AOP.4.000379.

[ref2] Donzella V.; Sherwali A.; Flueckiger J.; Fard S. T.; Grist S. M.; Chrostowski L. Sub-Wavelength Grating Components for Integrated Optics Applications on SOI Chips. Opt. Express 2014, 22, 21037–21050. 10.1364/OE.22.021037. 25321304

[ref3] Amitai Y.; Shechter R.; Reinhorn S.; Friesem A. A. Unconventional Optical Elements for Information Storage, Processing and Communications; Springer: Netherlands, 2000; pp 61–72.

[ref4] Neshev D.; Aharonovich I. Optical Metasurfaces: New Generation Building Blocks for Multi-Functional Optics. Light: Sci. Appl. 2018, 7, 58 10.1038/s41377-018-0058-1. 30839584 PMC6113330

[ref5] Buralli D. A.; Morris G. M. Design of Diffractive Singlets for Monochromatic Imaging. Appl. Opt. 1991, 30, 2151–2158. 10.1364/AO.30.002151. 20700190

[ref6] Kildishev A. V.; Boltasseva A.; Shalaev V. M. Planar Photonics with Metasurfaces. Science 2013, 339, 1232009 10.1126/science.1232009. 23493714

[ref7] Banerji S.; Meem M.; Majumder A.; Vasquez F. G.; Sensale-Rodriguez B.; Menon R. Imaging with Flat Optics: Metalenses or Diffractive Lenses?. Optica 2019, 6, 805–810. 10.1364/OPTICA.6.000805.

[ref8] Khorasaninejad M.; Capasso F. Metalenses: Versatile Multifunctional Photonic Components. Science 2017, 358, eaam8100 10.1126/science.aam8100. 28982796

[ref9] Engelberg J.; Levy U. The Advantages of Metalenses Over Diffractive Lenses. Nat. Commun. 2020, 11, 1991 10.1038/s41467-020-15972-9. 32332770 PMC7181857

[ref10] d’Auria L.; Huignard J.; Roy A.; Spitz E. Photolithographic Fabrication of Thin Film Lenses. Opt. Commun. 1972, 5, 232–235. 10.1016/0030-4018(72)90086-7.

[ref11] Komar P.; Gȩbski M.; Czyszanowski T.; Dems M.; Wasiak M. Planar Focusing Reflectors Based on Monolithic High Contrast Gratings: Design Procedure and Comparison with Parabolic Mirrors. Opt. Express 2020, 28, 38745–38761. 10.1364/OE.404684. 33379437

[ref12] Gȩbski M.; Dems M.; Szerling A.; Motyka M.; Marona L.; Kruszka R.; Urbańczyk D.; Walczakowski M.; Pałka N.; Wójcik-Jedlińska A.; Wang Q. J.; Zhang D. H.; Bugajski M.; Wasiak M.; Czyszanowski T. Monolithic High-Index Contrast Grating: a Material Independent High-Reflectance VCSEL Mirror. Opt. Express 2015, 23, 11674–11686. 10.1364/OE.23.011674. 25969259

[ref13] Fattal D.; Li J.; Peng Z.; Fiorentino M.; Beausoleil R. G. Flat Dielectric Grating Reflectors with Focusing Abilities. Nat. Photonics 2010, 4, 466–470. 10.1038/nphoton.2010.116.

[ref14] Klemm A. B.; Stellinga D.; Martins E. R.; Lewis L.; Huyet G.; O’Faolain L.; Krauss T. F. Experimental High Numerical Aperture Focusing with High Contrast Gratings. Opt. Lett. 2013, 38, 3410–3413. 10.1364/OL.38.003410. 23988971

[ref15] Fang W.; Huang Y.; Duan X.; Liu K.; Fei J.; Ren X. High-Reflectivity High-Contrast Grating Focusing Reflector on Silicon-on-Insulator Wafer. Chin. Phys. B 2016, 25, 114213 10.1088/1674-1056/25/11/114213.

[ref16] Fang W.; Huang Y.; Fei J.; Duan X.; Liu K.; Ren X. Concentric Circular Focusing Reflector Realized Using High Index Contrast Gratings. Opt. Commun. 2017, 402, 572–576. 10.1016/j.optcom.2017.06.096.

[ref17] Chen Q.; Fang W.; Huang Y.; Duan X.; Liu K.; Sharawi M. S.; Ren X. Uni-Traveling-Carrier Photodetector with High-Contrast Grating Focusing-Reflection Mirrors. Appl. Phys. Express 2020, 13, 016503 10.7567/1882-0786/ab5b4a.

[ref18] Arbabi A.; Arbabi E.; Horie Y.; Kamali S. M.; Faraon A. Planar Metasurface Retroreflector. Nat. Photonics 2017, 11, 415–420. 10.1038/nphoton.2017.96.

[ref19] Lu F.; Sedgwick F. G.; Karagodsky V.; Chase C.; Chang-Hasnain C. J. Planar High-Numerical-Aperture Low-Loss Focusing Reflectors and Lenses Using Subwavelength High Contrast Gratings. Opt. Express 2010, 18, 12606–12614. 10.1364/OE.18.012606. 20588387

[ref20] Mateus C.; Huang M.; Chen L.; Chang-Hasnain C.; Suzuki Y. Broad-Band Mirror (1.12-1.62 μm) Using a Subwavelength Grating. IEEE Photonics Technol. Lett. 2004, 16, 1676–1678. 10.1109/LPT.2004.828514.

[ref21] Marciniak M.; Gȩbski M.; Dems M.; Haglund E.; Larsson A.; Riaziat M.; Lott J. A.; Czyszanowski T. Optimal Parameters of Monolithic High-Contrast Grating Mirrors. Opt. Lett. 2016, 41, 3495–3498. 10.1364/OL.41.003495. 27472602

[ref22] Sreenivasan S. V. Nanoimprint Lithography Steppers for Volume Fabrication of Leading-Edge Semiconductor Integrated Circuits. Microsyst. Nanoeng. 2017, 3, 17075 10.1038/micronano.2017.75. 31057889 PMC6445000

[ref23] Kim S. Coherent Nonlinear Phenomena in Subwavelength-Grating Based Microcavities. Ph.D. Thesis, The University of Michigan, 2019.

[ref24] Dems M. Modelling of High-Contrast Grating Mirrors. The Impact of Imperfections on Their Performance in VCSELs. Opto-Electron. Rev. 2011, 19, 340–345. 10.2478/s11772-011-0027-1.

[ref25] Johnston T. F. Beam Propagation (M^2^) Measurement Made As Easy As It Gets: The Four-cuts Method. Appl. Opt. 1998, 37, 4840–4850. 10.1364/AO.37.004840. 18285945

