# Supplementary material for: Experimental Demonstration of Light Focusing Enabled by Monolithic High-Contrast Grating Mirrors
Source: ACS Appl Mater Interfaces. 2021 May 19;13(21):25533–9. doi: 10.1021/acsami.1c04871 (PMC8289169; doi:10.1021/acsami.1c04871)
Supplement: Supplementary file 2 — am1c04871_si_002.pdf [file am1c04871_si_002.pdf]

Experimental Demonstration of Light Focusing Enabled by Monolithic High-Contrast Grating Mirrors – Supporting Information

Paulina Komar,[*,†] Marcin Gębski,[†,‡] James A. Lott,[‡] Tomasz Czyszanowski,[†] and Michał Wasiak[†]

†*Institute of Physics, Lodz University of Technology, Wólczańska 219, 90-924 Łódź, Poland.*

‡*Institute of Solid State Physics and Center of Nanophotonics, Technical University Berlin, Hardenbergstraße 36, 10623 Berlin, Germany.*

E-mail: paulina.komar@p.lodz.pl

1 TE polarized light incident on the focusing grating mirror designed for the TM polarization

According to our simulations shown in Fig. S1(a), no light focusing is expected to occur if the focusing grating mirror designed for the TM polarization is illuminated with a TE polarized light. As a reminder, TE (TM) polarization means that the electric field is parallel (perpendicular) to the stripes of the grating. In turn, the experiment yields a map presented in Fig. S1(b), which shows that the focusing is not completely absent, however, the focal point is not as well-defined as in the case of TM polarization. An increased light intensity extends from $z = 200$ µm to $z = 550$ µm. Moreover, the maximum light intensity observed

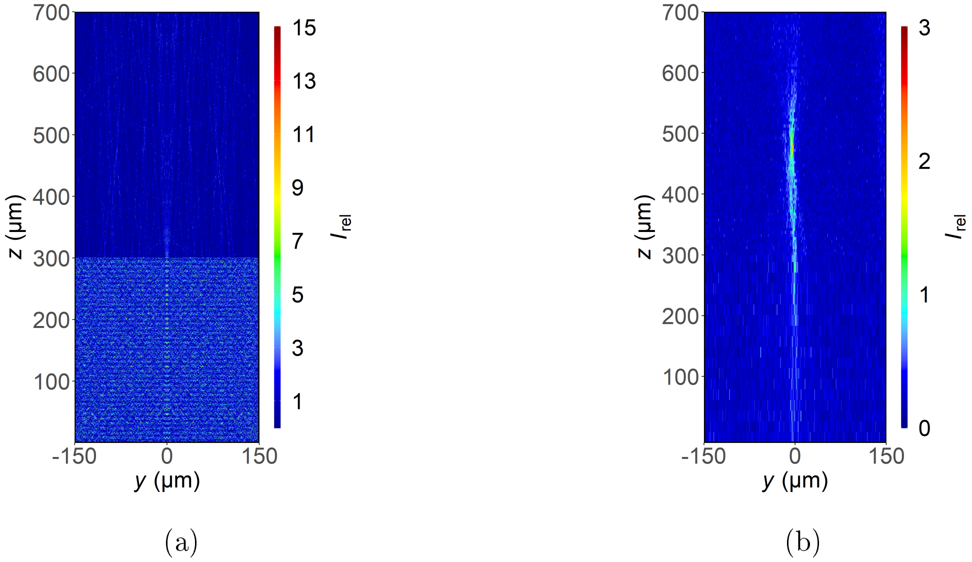

Figure S1: (a) Simulated and (b) measured maps of the reflected light intensity relative to the incident light intensity acquired for the focusing grating mirror that was designed for the TM polarization, on which the TE polarized light was incident.

for the TE polarization is approximately 15 times smaller than that determined for the TM polarization. This residual focusing is probably due to non-perfect polarization of the incident light or arises from an asymmetry of the grating due to non-ideal joint of the writing fields in the lithography process.

2 Linearity of the camera matrix

In order to determine the linearity of the camera matrix we used a setup shown in Fig. S2. The aim of this experiment was to determine whether the pixel RGB values stored in the RAW images are linearly proportional to the actual optical power. As a light source we used a 970-nm LED instead of the 980-nm VCSEL that was used in the experiments described in the main body of the article. Using an LED we were able to avoid the speckle pattern that occurs for lasers and we obtained greater illumination uniformity on the camera matrix. As the wavelengths are comparable and the spectral width of the LED is greater than 10 nm, we expect similar results for a wavelength of 980 nm. In front of the light source we positioned a variable neutral density filter which was used to vary the light attenuation in a well-controlled

and continuous manner. The light transmitted through the filter was divided into two rays by a 8%/92% beam splitter (8% of the intensity was directed to the camera and 92% of the intensity reached an optical power meter).

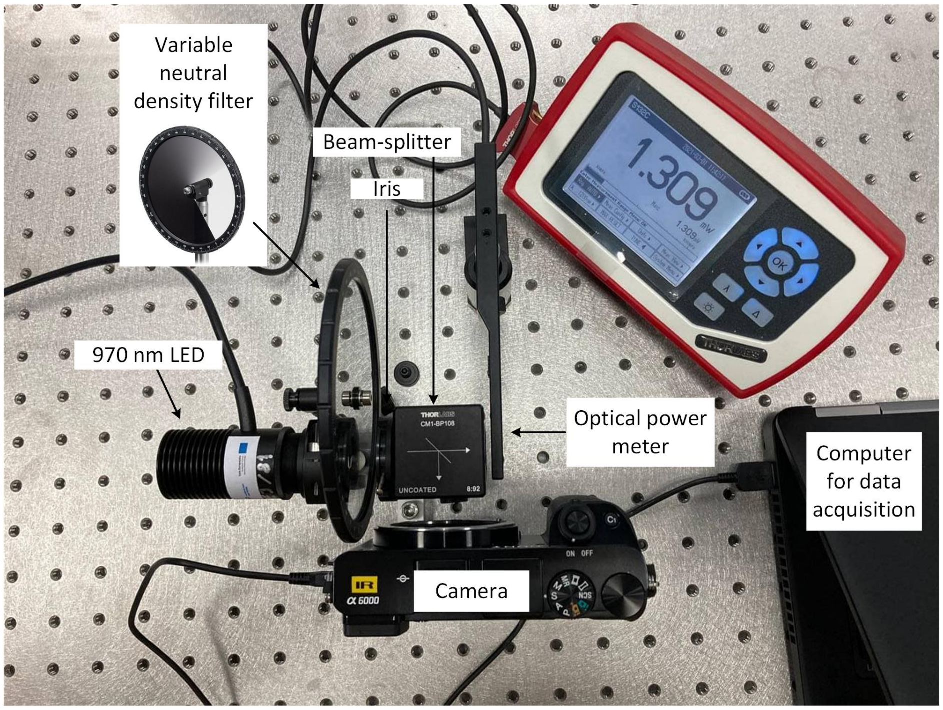

Figure S2: A photograph of the setup used to determine the range of the linearity of the camera matrix.

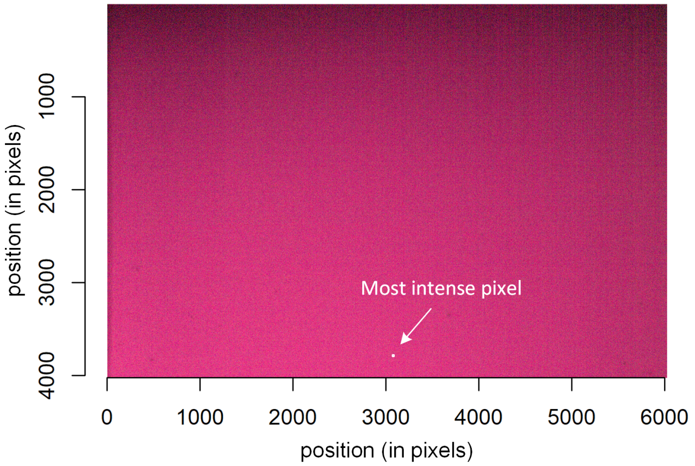

Figure S3: A photograph taken by a camera that was arranged in the setup shown in Fig. S2. The white point indicates the most intense pixel on the photograph.

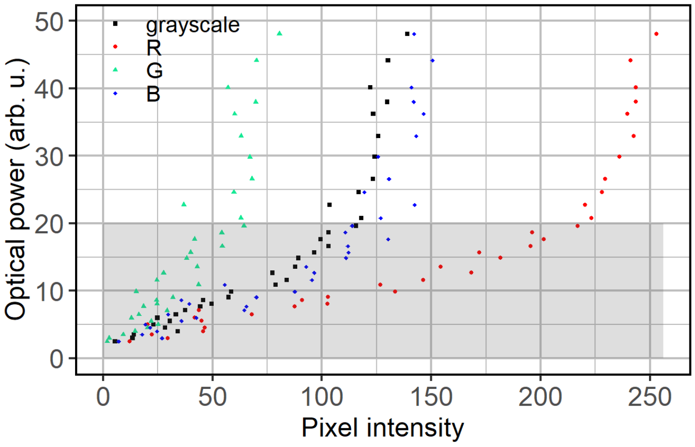

Figure S4: A graph showing what are the grayscale pixel intensity and all color component (red, green, and blue) intensities of the brightest pixel (indicated in Fig. S3) that correspond to the measured optical power.

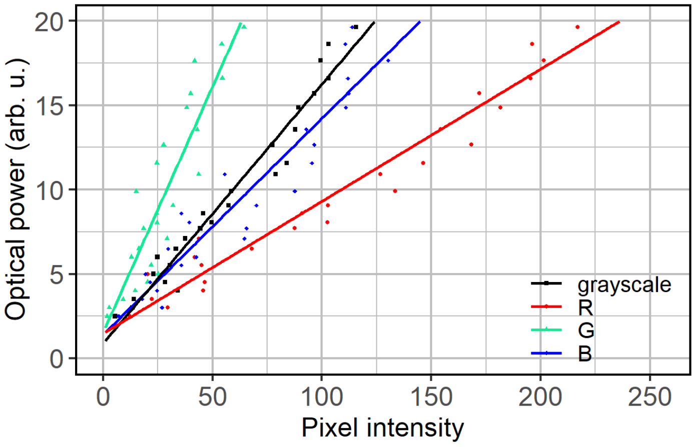

Figure S5: Magnification of the gray-shaded area from Fig. S4. The lines are fits to the data points.

We took a series of images for variable light attenuation (i.e. variable rotation angles of the filter). For every position of the filter we recorded also the optical power measured by the power meter. In the first step of the analysis we selected the most intense pixel on the photographs (indicated with a white dot and an arrow in Fig. S3). To avoid the hot-pixel effect, in fact our analysis window was not a single pixel, but the most intense pixel with ± 3 pixels adjacent to it in both directions, what corresponds to a group of 7×7 of pixels. We

analyzed the RAW images using R language with *imager* and *magick* libraries that allow to obtain red, green, and blue components of every pixel as a floating-point number from the range between 0 and 256. After analyzing the entire series of images, we prepared a plot on which we assign the mean pixel intensities of the previously selected 49 pixels to the measured optical powers. The plot in Fig. S4 presents the intensities of all the color components (R – red, G – green, B – blue, drawn in corresponding colors) as well as a grayscale intensity (shown in black). One can distinguish two regions in this graph, namely for optical powers smaller (gray-shaded) and larger than 20. In both of these regions the data may be approximated by linear dependencies. The gray-shaded area was also magnified in Fig. S5, where the solid lines show linear fits to the data points. Based on this analysis one can say that when the wavelength of 980 nm is incident on the camera matrix, the intensities of pixels are linearly proportional to the optical power, provided that the pixel intensities are smaller than 124 for grayscale intensity, 236 for the red component, 63 for the green component, and 145 for the blue component. These numbers were determined based on the linear fits as the pixel intensities for which the optical power is 20. A similar analysis of the linearity performed for a number of randomly distributed positions on the entire matrix gives consistent results.

To determine what is the range of pixel intensities in the analysis shown in Fig. 4 in the main body of the article, it is necessary to go back to the photograph illustrating the light intensity at the focal point of the focusing grating mirror. The photograph and the corresponding light intensity profiles in pixel intensities (grayscale and RGB) are shown in Fig. S6. The peak intensities are 125 for grayscale, 218 for R, 74 for G, and 158 for B. To assure that the light intensities shown in Fig. 4(g) in the article are correctly determined, the peak intensity of the profile in grayscale should be in the range determined in Fig. S5, i.e. smaller than or equal to 124. An excess of the linearity threshold is minor and should not affect the results presented Fig. 4 in the main body of the article, or could be only slightly underestimated.

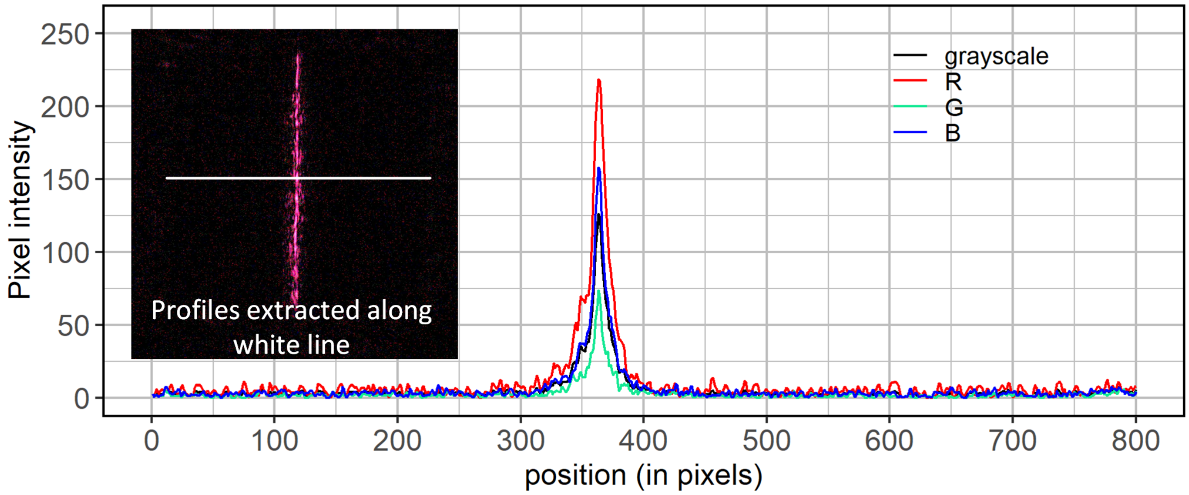

Figure S6: Red, green, blue, and grayscale pixel intensities of the mean profile extracted from the photograph of the light intensity at the focal point of the investigated focusing grating mirror. The photograph is shown in the inset and the procedure of the profile extraction is described in the main body of the article. The pixel intensities are plotted as a function of the position on the photograph that is expressed in pixels.

The presented analysis proves that for a wavelength of 980 nm there exists a certain range of pixel intensities at which the pixel intensities are linearly proportional to the optical power of the light incident on the matrix. In our experiment, the range of considered pixel intensities is at the limit of the linearity range, but we do not exceed it by more than 1%.

3 Resolution

The images observed on the camera matrix are influenced by the limited resolution of the optics (microscope objective) and the matrix itself. In order to take this effect into account, we performed the following theoretical 1D analysis.

Mathematically, the final image J can be expressed as a convolution of the observed pattern P and a function describing the limited resolution of the system ϱ:

$$J(y) = (P * \varrho)(y) = \int_{-\infty}^{\infty} P(\xi)\varrho(y-\xi)\, d\xi \tag{1}$$

Function ϱ itself could be expressed as a convolution of the functions describing the impact of the objective and the impact of the pixel structure of the matrix. The diffraction effect created by the objective can be approximated by a Gaussian distribution with the standard deviation $\sigma_{\mathrm{o}} = 0.21\lambda/\mathrm{NA}$.[1] The matrix-related resolution is related to the discrete pixels on the matrix and unknown interpolation algorithms used by the manufacturer. We can estimate the variance of this function as $\sigma_{\mathrm{m}}^2 = (w/\mathrm{mag})^2/12$, where w is the pixel width and mag is the magnification of the objective. This is simply a formula for variance of a rectangular distribution, so we underestimate σ_{m} by neglecting the impact of the interpolation. However, as it will be shown later, the impact of the camera resolution is negligible anyway. After substituting the parameters of our set-up ($\lambda = 0.98\,\mu\mathrm{m}, \mathrm{NA} = 0.28, w = 3.92\,\mu\mathrm{m}, \mathrm{mag} = 10$) we obtain:

$$\sigma_{\mathrm{o}} = 0.735\,\mu\mathrm{m} \qquad \sigma_{\mathrm{m}} = 0.113\,\mu\mathrm{m} \tag{2}$$

The variance σ^2 of the distribution ϱ is simply a sum of the two variances, so the resulting standard deviation is:

$$\sigma = \sqrt{\sigma_{\mathrm{o}}^2 + \sigma_{\mathrm{m}}^2} \approx 0.744\,\mu\mathrm{m} \tag{3}$$

Whatever the distribution of the matrix-related part of the convolution is, the distribution of ϱ must be close to a Gaussian distribution because of the difference in the two variances. Therefore, we assume that ϱ is also a Gaussian distribution with the standard deviation σ. Provided that our estimate of σ_{m} is not a few times too low, in our system, the objective is the element limiting the resolution and the impact of the camera is practically negligible.

References

(1) Zhang, B.; Zerubia, J.; Olivo-Marin, J.-C. Gaussian Approximations of Fluorescence Microscope Point-Spread Function Models. *Appl. Opt.* **2007**, *46*, 1819–1829.
